# A string theoretic derivation of gibbons-hawking entropy

**DOI:** 10.1007/s10714-025-03446-6

**Published:** 2025-08-02

**Authors:** Gia Dvali

**Affiliations:** 1 Arnold Sommerfeld Center, Ludwig-Maximilians-University, Munich, Germany; 2 Max-Planck-Institute for Physics, Munich, Germany

**Keywords:** String theory, de Sitter, Entropy, Black holes, D-branes, Inflation

## Abstract

We describe an attempt of string theoretic derivation of the Gibbons-Hawking entropy. Despite not admitting a de Sitter vacuum, the string theory, by the power of open-close correspondence, captures the Gibbons-Hawking entropy as the entropy of Chan-Paton species on a de Sitter-like state obtained via *D*-branes. Moreover, this derivation sheds a new light at the origin of the area-form, since the equality takes place for a critical ’t Hooft coupling for which the species entropy of open strings saturates the area-law unitarity bound.

## Introduction

Some time ago [1] Gibbons and Hawking pointed out that de Sitter space possesses a microstate entropy that scales as the area of the horizon,

1.1 $$\begin{aligned} S_{GH} \, \sim \, (R M_P)^2 \,, \end{aligned}$$

where *R* is the de Sitter curvature radius and $M_P$ is the Planck mass. This expression is very similar to the Bekenstein-Hawking entropy of a black hole of equal radius [2, 3].

While there is a full consensus about the correctness of $S_{GH}$ entropy, there exist no commonly accepted microscopic explanation for it. It is natural to ask whether we can gain some insight within string theoretic framework.

In this respect the situation with de Sitter is rather peculiar and goes in contrast with black holes. Namely, there exist a well-known string theoretic derivation of Bekenstein entropy of an extremal black hole by Strominger and Vafa [4].

There also exist other stringy handles on black hole entropy. For example, more recently, it became evident that the Bekenstein-Hawking entropy can be read out from the *S*-matrix elements of multi-graviton and multi-closed string scattering processes at trans-Planckian center of mass energies [5, 6]. Correspondingly, such processes can be interpreted as the *S*-matrix processes describing a black hole formation at trans-Planckian energies [7–9], in which the black hole is resolved as a multi-graviton coherent state [10].

At the same time, we are not aware of any string theoretic derivation of Gibbons-Hawking entropy. The goal of the present paper is to fill this omission. We shall base our discussion on the arguments and the construction already provided in [11], where it was shown that via open-closed duality the string theory carries a non-trivial information about the Gibbons-Hawking entropy of de Sitter.

Let us fist notice that there are some fundamental differences between the ways in which black holes and de Sitter are treated by the string theory. First, no de Sitter vacuum exist in string theory. Its absence can be understood as a result of *S*-matrix on which the formulation of string theory is intrinsically based [16]. Any eternally-inflating de Sitter-like universe makes a definition of *S*-matrix impossible. To our opinion, this feature is an intrinsic source of various issues with formulation of quantum gravity in de Sitter discussed by Witten [12].

The absence of de Sitter *S*-matrix justifies the earlier proposals about incompatibility of de Sitter with quantum gravity [13–15]. It also fully resonates with the so-called de Sitter conjecture [17] and, in the present context, even more closely with the trans-Planckian censorship conjecture [18].

In contrast, as already remarked, black holes are in no conflict with *S*-matrix. For example, the unstable, non-extremal, black holes can be described as intermediate finite-lifetime resonances formed in an *S*-matrix process with trans-Planckian center of mass energies. Alternatively, the stable black holes, such as the extremal ones, can be viewed as legitimate asymptotic *S*-matrix states.

None of the above is possible for de Sitter. Unlike a black hole, the de Sitter is an eternally-inflating state with no possibility of defining the *S*-matrix [16]. This difference can also be understood from the fact that black holes can be formed in a collision process of in-states without any need of a vacuum energy. In contrast, de Sitter necessarily requires an uniform classical source in form of a cosmological constant.

However, the urgency for string-theoretic understanding of Gibbons-Hawking entropy transcends the question of the de Sitter vacuum. The reason is that the Gibbons-Hawking entropy is expected to be an intrinsic property of any slow-roll inflationary Hubble patch even with a moderate number of e-foldings, not conflicting with the existence of an asymptotic *S*-matrix vacuum.

In the present note, refining the earlier work [11], we shall show that, despite not supporting a de Sitter vacuum, the string theory nevertheless captures the origin of Gibbons-Hawking entropy thanks to the power of open-closed correspondence. In the above paper it was observed that in the regime in which the system admits a de Sitter like state, the Gibbons-Hawking entropy is accounted by the species entropy of the open-string Chan-Paton modes. We shall essentially repeat the construction of [11] and attempt to give it a form of a full-fledged derivation.

## A string theoretic construct

Before moving to actual derivation, we make some preparatory remarks and define some useful quantities. The two important parameters are: the species scale $M_{sp}$ and the species entropy $S_{sp}$.

The species scale sets an absolute cutoff on any *d*-dimensional theory of gravity propagating $N_{sp} \gg 1$ particle species and is given by [19–26, 11],

2.1 $$\begin{aligned} M_{sp} = \frac{M_{d}}{N_{sp}^{\frac{1}{d-2}}} \,, \end{aligned}$$

where $M_d$ is the *d*-dimensional Planck mass. The scale $1/M_{ps}$ is the lower bound on the radius of a black hole beyond which the extrapolation of semi-classical features is impossible. For example, a would-be black hole of a smaller size cannot maintain the thermal Hawking radiation, or else, such a black hole would explode in less than the light-crossing time, which would-be an absurd.

We must note that the species scale has perturbative manifestations [27, 28], however the power of the above black hole arguments is that they are fully non-perturbative and therefore are insensitive to a possible fine-tuning or a resummation of the series. More recent discussions of various non-perturbative manifestations of the species scale were given in [29–32].

Another important concept, on which we shall rely heavily, is the “species entropy", $S_{sp}$, which measures the capacity of the system to store information in the microstates of species [22, 24, 26, 11]. For the species that are approximately degenerate in mass, in a state with total occupation number *N*, the species entropy is given by the log of the binomial coeficient [26]:

2.2 $$\begin{aligned} S_{sp}= &   \ln \begin{pmatrix} N_{sp} + N \\ N_{sp} \end{pmatrix} \nonumber \\\simeq &   N_{sp} \ln ( (1+ N_{sp}/N)^{N/N_{sp}} (1+N/N_{sp})) \,, \end{aligned}$$

where in the last expression we used Stirling’s approximation. As shown in [26], the saturation of species entropy takes place for $N \sim N_{sp}$, which gives,

2.3 $$\begin{aligned} S_{sp} \sim N_{sp} \,. \end{aligned}$$

The species entropy gives an alternative justification to the statement that the cutoff is given by the species scale (2.1). Indeed, it has been shown [22, 24, 26] that for a would-be black hole smaller than the species scale, $1/M_{sp}$, the species entropy would exceed the Bekenstein-Hawking entropy, which is impossible.

In our derivation, via open-closed correspondence, the Gibbons-Hawking entropy shall emerge as the species entropy of the open-strings [11].

We start with an attempt of producing a de Sitter like state in string theory. We shall use the standard (anti)*D*-brane uplifting mechanism put forward in [33–35]. In fact, we do not have much alternatives since, as already realized in the above work, *D*-branes are the only known negative pressure sources in string theory.

The construction can be given in various string dimensions. However, in order to avoid the secondary issues with technicalities of compactification, we shall work in type *II*B in ten dimensions.

As usual, the two fundamental parameters are the string coupling, $g_s$, and the string scale, $M_s$. The ten-dimensional Planck mass, $M_{10}$, is expressed via $M_s$ and $g_s$ as,

2.4 $$\begin{aligned} M_{10}^8 = \frac{M_s^8}{g_s^2} \,. \end{aligned}$$

Throughout the paper we shall omit the irrelevant numerical factors which can be easily restored.

Following [11], we shall prepare a space-filling background with *n* coincident $D_9$-branes and *n* anti-$\bar{D}_9$-branes. The world-volume (open string) physics of this system is rather well-understood [39]. The open string zero modes produce $N_{sp} \sim n^2$ light Chan-Paton species which transform under the $U(n)\times U(n)$ gauge symmetry.

As discussed in [24], such a background has associated ten-dimensional species scale

2.5 $$\begin{aligned} M_{sp} \, = \, \frac{M_{10}}{n^{1/4}} \, = \frac{M_s}{(ng_s)^{\frac{1}{4}}}\,, \end{aligned}$$

and the corresponding species entropy (2.2), which at the saturation point takes the value (2.3),

2.6 $$\begin{aligned} S_{sp} = n^2 \,. \end{aligned}$$

This entropy reflects the existence of microstates with different excitation patterns of the Chan-Paton species.

We shall be working in the ’t Hooft limit [36] of large *n* and weak string coupling:

2.7 $$\begin{aligned} n \rightarrow \infty \,,~ g_s \rightarrow 0\,, \end{aligned}$$

while keeping the ’t Hooft coupling, $(ng_s)$, as well as the string scale, $M_s$, finite. We shall use the ’t Hooft coupling as a control parameter, and shall approach the critical value, $(ng_s) = 1$, from below.

Notice that this background generates a no-trivial scalar potential for the dilaton, thereby, likely making it time-dependent. We shall not enter in the discussion of dilaton-stabilization^1^. Instead, we shall limit ourselves by restricting the time-scale of its potential evolution. The curvature scale of the dilaton effective potential is set by the quantity,

2.8 $$\begin{aligned} M_s^2 (ng_s)\,. \end{aligned}$$

This makes the potential time-dependence of the dilaton sufficiently slow for our arguments to go through. We thus assume that on the time-scales of our interest, dilaton is approximately frozen.

However, the background contains an open-string tachyon, with negative mass$^2$ of order $m^2_t \sim - M_s^2$. This reflects the instability towards mutual annihilation of *D*-branes and anti-*D*-branes. This process can be described as the tachyon condensation [40]. The classification is given in terms of *K*-theory [45].

When tachyon condenses, the Chan-Paton species become massive. A part of them gains the mass via the Higgs effect. Others become massive non-perturbatively. This is also obvious from the fact that the closed-string vacuum cannot support the open string excitations without *D*-branes.

Now, the idea of “brane inflation" scenario [33–35] is that, prior to tachyon condensation, the energy density coming from *D*-brane tensions acts as vacuum energy, creating a temporary de Sitter-like inflationary state. The tachyon condensation then provides a graceful exit from inflation. The duration of the inflationary state depends on the details of constructions in various dimensions to which we shall not enter.

We shall rather focus on the state prior to tachyon condensation in which the tachyon is carefully tuned in the position of unstable equilibrium, similarly to a Higgs field on top of a “Mexican hat" potential.

For a moment, let us mentally freeze the tachyon. Then, due to a positive vacuum energy density,

2.9 $$\begin{aligned} \Lambda _{10} = n \frac{M_s^{10}}{g_s} \,, \end{aligned}$$

coming from the collective tension of *D*-branes, classically, the system would produce a ten-dimensional de Sitter metric. The corresponding de Sitter curvature radius would be^2^

2.10 $$\begin{aligned} R \, = \, \sqrt{M_{10}^8/\Lambda _{10}} = \frac{1}{\sqrt{ng_s} M_s} \,. \end{aligned}$$

Therefore, the Gibbons-Hawking entropy would be equal to,

2.11 $$\begin{aligned} S_{GH} = (R M_{10})^8 = \frac{1}{g_s^2(ng_s)^4} \,. \end{aligned}$$

Thus, in a state with artificially frozen tachyon, the system would posses two types of entropies: *1)* The gravitational Gibbons-Hawking entropy (2.11), coming from closed strings; and *2)* The Chan-Paton species entropy (2.6), coming from open strings. Their ratio scales as,

2.12 $$\begin{aligned} \frac{S_{sp}}{S_{GH}} = (ng_s)^6 \,. \end{aligned}$$

This may look disappointing, since keeping in mind the open-closed correspondence, one could have hoped that the two entropies would come out equal. However, the situation is quite the contrary.

In reality, none of the entropies are real, since the system is unstable on the time-scale less than the Hubble time. In order to talk about entropies, we must first reach the state in which the system is stabilized, at least for one Hubble time. Only in such a state it makes sense to compare the two entropies.

Now, following [11], we can try to self-consistently stabilize the tachyon using a general mechanism discussed earlier in [34]. We proceed in the following way. Instead of Chan-Paton vacuum, we consider a state in which the species are excited. Such excitations generate a positive contribution into the mass$^2$ of the tachyon field and can potentially stabilize it.

In fact, in a would-be de Sitter, the Chan-Paton species would be automatically excited due to the Gibbons-Hawking temperature of de Sitter,

2.13 $$\begin{aligned} T_{GH} = \frac{1}{R} \,. \end{aligned}$$

Thus, we can proceed in the following manner. We can assume that tachyon has been stabilized and check the self-consistency of this assumption by verifying the thermal contribution to its mass from the Chan-Paton species.

In a would-be de Sitter, the effective mass-square of the tachyon would receive the following positive contribution from the coupling to the thermal bath of Chan-Paton species at temperature $T_{GH}$^3^

2.14 $$\begin{aligned} \Delta m^2_{t} = \frac{(ng_s)^2}{R^2} = (ng_s)^3 M_s^2 \,. \end{aligned}$$

This contribution would balance the zero-temperature negative mass-square of the tachyon, provided the parameters satisfy,

2.15 $$\begin{aligned} (ng_s) \sim 1 \,. \end{aligned}$$

In this case the stack of branes could be stabilized.

This mechanism of potential stabilization of *D*-branes on top of each other was previously considered in [34] and can be viewed as the effect of restoration of $U(n)\times U(n)$ symmetry at non-zero temperature.

The remarkable thing is that at the critical point (2.15) the Gibbons-Hawking entropy becomes equal to the entropy of species (2.12), as this would be expected from the open-closed correspondence.

The presented reasoning can be viewed as the microscopic understanding of Gibbons-Hawking entropy in terms of the entropy of Chan-Paton species.

One may complain that there is a potential loophole because one could seemingly violate the equality between the two entropies by taking

2.16 $$\begin{aligned} (n g_s) \gg 1\,. \end{aligned}$$

This is however not possible, since it would put our calculation out of the domain of validity. This is signalled by several factors.

First, it was already noticed in [24] that for (2.16), the ten-dimensional species scale (2.5) which sets the absolute cutoff of effective low energy theory, would drop below the string scale $M_s$. This is not possible, since the cutoff in string theory is the string scale.

Simultaneously, the de Sitter curvature radius *R* (2.10) would become shorter than the string length $1/M_s$. This would signal that any geometric notion of de Sitter becomes non-existent.

In particular, the Gibbons-Hawking temperature (2.13) of a would-be de Sitter state exceeds the string scale and the Hagedorn effects [37] must be taken into account [38]. At this point various stringy effects, such as Atick-Witten phase transition [42], also enter^4^.

The important message that we deduce from our analysis is that in the unique regime (2.15), in which the system can potentially deliver a de Sitter-like state, the Gibbons-Hawking entropy (2.11) is understood as the species entropy (2.2) of the Chan-Paton species. At the critical point (2.15), the two entropies are equal and they satisfy:

2.17 $$\begin{aligned} S_{GH} = S_{sp} = n^2 = \frac{1}{g_s^2} \,. \end{aligned}$$

Thus, by the power of open-closed correspondence, string theory offers a microscopic understanding of Gibbons-Hawking entropy in terms of species entropy of open strings.

## Species origin of the area-law

With the equation (2.17) our task of string theoretic derivation of Gibbons-Hawking entropy is complete. What comes next is the interpretational part. Although this interpretation changes nothing in the above derivation, it is nevertheless physically important, as it opens up a new view on open-closed correspondence.

Namely, we would like to give some physical intuition to a “miracle" of obtaining an intrinsically-geometric area-form of Gibbons-Hawking entropy (2.11), from a seemingly-non-geometric concept of open string species entropy (2.2).

In fact, the story goes beyond the usual view of “holography" in which a geometric area-count of the entropy is an exclusive feature of the gravitational part of the theory (for a review, see [46]).

Instead, as we shall see, both entropies, $S_{sp}$ and $S_{GH}$, independently have very clear geometric area-forms. This feature is fundamentally linked with the criticality of the ’t Hooft coupling (2.15). Moreover, at the same time, for the critical coupling the two entropies are equal (2.17). That is, in certain sense, the open-closed correspondence is responsible not for the area-forms of the two entropies *per se*, but for their equalities at the critical ’t Hooft coupling (2.15).

The key point is that, irrespective of gravity, the species microstate entropy does have a geometric meaning in form of the following universal bound on the microstate degeneracy [26]:

3.1 $$\begin{aligned} S_{max} = (Rf)^{d-2} \,. \end{aligned}$$

This bound implies that, within the validity of a given description, the maximal microstate entropy attained by a *d*-dimensional system of a radius *R* is equal to its surface area in units of the scale of a spontaneously broken Poincare symmetry, *f*.

The validity domain is defined in the usual quantum field theoretic (or string theoretic) sense as the requirement of a weak coupling of the relevant degrees of freedom. In particular, the saturation of the bound (3.1) is correlated with the saturation of unitarity by a set of scattering amplitudes [26].

The bound (3.1) is universal and is independent of gravity, as long as the system can be defined as a state on an asymptotic vacuum of Minkowski. In the present case, this condition is satisfied, since our de Sitter-like state is not eternal and is going to asymptote into a ten-dimensional Minkowski vacuum. We can therefore classify the states according to the Poincare symmetry of that vacuum.

Here, we shall not enter in the justification of the bound (3.1) for generic systems, which can be found in [26] and in many related papers (for an incomplete list, see, [47–53]). Instead, we shall limit ourselves by providing an intuition for the formula (3.1) specifically for the species entropy, again following [26].

Let us imagine a *d*-dimensional system of radius *R* with a total occupation number *N* of massless particle species. Such a system breaks the Poincare symmetry at the scale

3.2 $$\begin{aligned} f = \frac{N^{\frac{1}{d-2}}}{R}\,. \end{aligned}$$

This is easy to understand from the fact that the quantity 1/*N* is the dimensionless coupling of the corresponding Goldstone mode.

At the same time, the species entropy (2.2) at the saturation point is given by $N = N_{sp}$. This immediately shows that (2.3) is given by (3.1). Thus, even in the flat space, the species entropy, at its saturation point, carries a very clear area-law meaning.

Applied to our *D*-brane construction, it is easy to see that at the critical point (2.15) the scale of spontaneous breaking of the ten-dimensional Poincare invariance is the ten-dimensional Planck scale,

3.3 $$\begin{aligned} f = \frac{n^{\frac{1}{4}}}{R} = M_{10}\,. \end{aligned}$$

Correspondingly, the entropy of Chan-Paton species (2.6), which was already shown to be equal to Gibbons-Hawking entropy (2.17), at the same time, saturates the area-law bound (3.1) with $f = M_{10}$. Obviously, for $f= M_{10}$, the equation (3.1) matches the general Gibbons-Hawking expression (2.11).

We thus see that the open-closed correspondence allows to understand the Gibbons-Hawking entropy, and most importantly its area-form, as the saturation of the universal bound (3.1) by the Chan-Paton species entropy (2.6) at the critical ’t Hooft coupling (2.15).

## Some implications

Here we briefly discuss some implications of our results.

### Glimpses of de sitter memory burden

The above construction also allows us to catch glimpses of a de Sitter “memory-burden" effect [55]. The essence of the memory burden phenomenon [54–56, 59] is that the load of information tends to stabilize the system. The effect is especially prominent in systems of maximal capacity of information (memory) storage, in particular, the systems that saturate the bound (3.1) on the microstate degeneracy, so-called “saturons".

In such a system the information is stored in a set of degrees of freedom called “memory modes". Lower is the excitation energy cost of the memory mode, more efficient is the information storage capacity of the system.

The special feature of enhanced memory systems is that they possess a critical state in which many species of nearly-gapless memory modes emerge. This criticality can be parameterized by a control order parameter called a “master mode".

The load of information corresponds to an excitation pattern of the memory modes. This stabilizes the system in the state in which these modes are least costly in energy.

Since the effect is universally present in systems of high microstate degeneracy, it is expected to be operative in black holes [54, 56–58] and in de Sitter [55].

In case of a black hole, the memory burden is expected to slow down the decay after emission of about a half of the mass. The localized objects such as black holes [54, 56, 59] or solitons [52, 59], when burdened by memory, are relatively straightforward to be visualized.

However, the analogous question for de Sitter is more profound [55]. In particular, this is the case due to absence of an external observer.

In the present construction the meaning of the memory burden effect in a de Sitter-like state becomes rather explicit. Indeed, the memory modes are the Chan-Paton species, whereas tachyon is a master mode. When tachyon is on top of the Mexican hat, the memory modes are gapless. If tachyon condenses, system moves to the closed-string vacuum and the memory modes gain large mass gaps. For excited memory modes, this would be very costly in energy. Correspondingly, the excitations of Chan-Paton modes tend to create an energy barrier and stabilize the tachyon on top of the hill.

In this way, the string theory allows us to catch a glimpse of a de Sitter burdened by memory: it is a state at critical ’t Hooft coupling (2.15) with quantum stringy effects becoming as important as the classical ones.

### Quantum breaking of de sitter

From the *S*-matrix perspective [16], the de Sitter is expected to be describable as a coherent state of gravitons constructed on top of an asymptotic *S*-matrix vacuum of Minkowski [13–15] (for a BRST-invariant construction, see [60])^5^. This picture comes with an accompanying phenomenon of “quantum-breaking". Its essence is that the quantum evolution of the coherent state of gravitons, after a so-called quantum break-time, $t_Q$, fully departs from the classical one.

In [13] the quantum break-time from the inner entanglement of the graviton state was suggested to be $t_Q \sim R \ln (S_{GH})$. This is very similar to the time-scale given by the trans-Planckian censorship conjecture [18]. For the present stringy construction $t_Q$ translates as [16] $t_Q \sim R \ln (1/g_s^2)$. Needless to say, a more precise stringy computation of the log-factor would be highly informative.

## Outlook

In this note we offered a string theoretic view at the microscopic origin of the Gibbons-Hawking entropy. In the limit of critical ’t Hooft coupling in which a set of $D-\bar{D}$-branes delivers a de Sitter-like state, the entropy of open string Chan-Paton species reproduces the gravitational Gibbons-Hawking entropy. The crux of it all is in universal features of species entropy as well as in open-close correspondence of string theory. This allows for understanding of intrinsically gravitational entropy of closed strings in terms of non-gravitational entropy of the Chan-Patton species.

Remarkably, the underlying physics of this connection goes beyond the ordinary view on “holography" in which the geometric area-form is the exceptional property of only one side of the description. Instead, the non-gravitational species entropy, $S_{sp}$, at the saturation of unitarity, independently, has the area-form (3.1) controlled by the strength of spontaneous breaking of the Poincare symmetry. At the critical value (2.15) of the stringy ’t Hooft coupling, the two entropies become equal (2.17).

The following comments are in order. First, of course, we expect corrections from higher order operators [61]. It is important to understand how they correct the equation (2.17).

Secondly, the fact that our construction is valid up to a critical coupling (2.15), prompts the question whether an analog of Horowitz-Polchinski string-black hole correspondence [62] can be defined for the de Sitter case [61]. In certain sense, the presented construction indicates a path in this direction via providing a correspondence between the entropies of open-strings and Gibbons-Hawking.

Finally, it would be proper to understand a potential connection with AdS/CFT correspondence [63–65].

## Data Availability

No datasets were generated or analysed during the current study.

## Footnotes

1 We thank Cumrun Vafa for raising the question about dilaton stability.

2 Comparing with (2.8), it is clear that dilaton does not evolve faster that the Hubble time, which justifies the validity of our assumption.

3 Notice that a one-loop correction from stringy version of Coleman-Weinberg potential shall not overpower the thermal correction, if we use as a rough estimate the calculation of [41] with multiplicity of species $n^2$ and the coupling $g_s^2$.

4 Various discussion can be found in [43, 44], and references therein.

5 Interestingly, this picture passes an immediate consistency test of reproducing the Gibbons-Hawking entropy (2.11) as a particular case of saturation of the bound (3.1). Indeed, since the asymptotic vacuum is Minkowski, the notion of spontaneous breaking of Poincare symmetry by the de Sitter coherent state is well-defined and the scale of breaking is the Planck mass $f = M_d$, which again reproduces (2.11) from (3.1).
